# **Corrosive Injury of the Upper Gastrointestinal Tract: Review of Surgical Management and Outcome in 14 Adult Cases**

**Published:** 2015-01

**Authors:** Mohammad Taghi Rajabi, Ghodratollah Maddah, Reza Bagheri, Mostafa Mehrabi, Hossein Shabahang, Farjad Lorestani

**Affiliations:** 1 *Endoscopic & Minimally Invasive Surgery Research Center, Ghaem Hospital, School of Medicine, Mashhad University of Medical Sciences, Mashhad, Iran.*; 2 *Cardio- Thoracic Surgery & Transplant Research Center, Imam Reza Hospital, School of Medicine, Mashhad University of Medical Sciences, Mashhad, Iran.*; 3 *Surgical Oncology Research Center, Imam Reza Hospital, School of Medicine, Mashhad University of Medical Sciences, Mashhad, Iran.*

**Keywords:** Caustic ingestion, Esophageal replacement, Esophageal stricture.

## Abstract

**Introduction:**

Caustic ingestion is responsible for a spectrum of upper gastrointestinal tract injury from self-limited to perforation. This study conducted to evaluate clinical characteristics as well as surgical outcomes in patients with caustic ingestion.

**Materials and Methods:**

Between Nov1993 to march 2011, 14 adults with a clinical evidence of corrosive ingestion were admitted into our institutions (Omid and Ghaem hospitals). Patients evaluated for etiology of erosion, location, type of surgery, morbidity and mortality after surgery.

**Results:**

14 patients (10men and 4 women) with a age range between18-53 years were evaluated. In 6 patients, the injury was accidental and in 8 patients ingestion was a suicide attempt. Ingested agent included nitric acid in 4 patients, hydrochloric acid in 7 patients, sulfuric acid in 2 patients and strong alkali in one patient. The location and extent of lesion varied included esophagus in 13 cases, stomach in 7 cases and the pharynx in 3 cases. Acute abdomen was developed In 2 patients and a procedure of total gasterectomy and blunt esophagectomy was performed. In the remaining patients, substernal esophageal bypass in 2 patients, esophageal resection and replacement surgery in 9 patients and gastroenterostomy in one patient performed to relieve esophageal stricture. Two patients died of mediastinitis after esophageal replacement surgery. Postoperative strictures were developed in 2 survived patients with hypopharyngeal reconstruction that was managed by per oral bougienage in one patient and KTP Laser and stenting in the other patient.

**Conclusion:**

Esophageal resection with replacement was safe and good technique for severe corrosive esophageal stricture with low mortality and morbidity.

## Introduction

Corrosive substances are those that cause destruction or damage to living tissue on contact, and may usually be grouped into acids or alkalis (1). Although children under the age of 5 years account for more than 80 percent of corrosive ingestion, and although almost all ingestions are accidental, in adults a suicide attempt or gesture is the usual explanation for the ingestion of a corrosive substance (2). 

Caustic ingestion can result in a number of injuries ranging in severity from minimal mucosal erythema to transmural necrosis of the esophagus and stomach with viscous perforation (3). After recovery from the initial injury, esophageal stricture may develop. This complication is the most important complication of corrosive damage to the esophagus (3,4) and, despite good initial management, approximately 5–12% of patients who ingest caustic substances develop esophageal stricture (5,6). 

The mainstay of treatment of established stricture has been graded dilatation at intervals of 10–14 days for 6 to 12 months after the initial insult or until fibrosis is no longer progressive (7).

Therapeutic dilatation should be commenced immediately if the presence of a stricture is confirmed. A strong predictor of poor outcome is time delay from ingestion to commencement of dilatation.

When repetitive dilatation fails to relieve symptoms or in cases where the patient refuses to undergo repeated dilatations, more aggressive techniques are indicated to reconstruct the conduit from the hypo- pharynx to the stomach (3,8). 

Surgical treatment is divided into emergency procedures and delayed reconstruction (4). Emergency surgery is indicated for hemorrhage, free perforation or peritonitis (9).

Over the past 18 years we have treated 14 adult patients who have ingested caustic agents. This report assesses our methods of handling patients referred to our centers (Ghaem and Omid hospitals Mashhad University of Medical Sciences) who have sustained caustic injuries to the upper gastrointestinal tract and development of stricture formation. 

## Materials and Methods

During an 18-year period from November 1993 to March 2011, 14 adults with clinical evidence of corrosive ingestion were treated at our institutions. 

All patients suffering serious caustic ingestion were hospitalized and received intravenous fluid, antibiotics and corticosteroids. Endoscopic evaluation was performed to evaluate the extent of injury.

Emergency surgery was performed if extensive gastric necrosis developed. These patients underwent total gastrectomy and blunt esophagectomy without thoracotomy with left, terminal esophagostomy and a duodenal closer with feeding jejunostomy. In stricture groups, definitive surgery including substernal esophageal bypass, esophageal resection and replacement and gastroenterostomy was performed if the dilatation was not successful. 

## Results

During the 18-year period, 14 patients (10 men and four women) with caustic ingestion were evaluated. The ages of the subjects ranged from 18 to 54 years, with an average of 26.4 years. The caustic ingestion was accidental in six patients and a suicidal attempt in eight patients. 

The corrosive agents were a strong acid in 13 cases (nitric acid in four, hydrochloric acid in seven and sulfuric acid in two cases). 

Liquid lye (potassium hydroxide) was responsible for only one case. The location and extent of the lesions varied, and included the esophagus in 13 cases, the stomach in seven cases, and the pharynx in three cases. 

Table 1 shows the characteristics of the patients included.

**Table 1:** Types of operative reconstruction for corrosive injuries

**Case**	**Age /sex**	Caustic Agent	**Upper Anastomosis**	**Mediastinal Route**	**Lower Anastomosis**
	27/M	Sulfuric acid	Right ileohypopharyngostomy via supra hyoid approach	Substernal	Cologastrostomy + gastrojejunostomy
2	54/M	**Nitric acid**	Left coloesophagostomy (antiperistatic)	Substernal	Coloesophagostomy + gastro jejunostomy
	41/M	Nitric acid	Esophagogastrectomy + left coloesophagostomy (antiperistatic)	Posterior	Colojejunostomy
4	19/F	Hydrochloric acid	Right ileohypopharyngostomy via transepigottic approach	Substernal	Cologastrostomy gastrojejunostomy
5	16/F	Hydrochloric acid	Gastroesophagostomy (gastric pull up)	Posterior	------------
6	18/M	Potassium Hydroxide	Gastroesophagostomy (gastric pull up)	Posterior	------------
7	24/M	Hydrochloric acid	Right ileoesophagostomy	Substernal	Colojejunostomy
8	29/M	Nitric acid	-----------	----------	Gastrojejunostomy + truncal vagotomy
9	24/M	Hydrochloric acid	Left colohypopharyngostomy (antiperistaltic) via suprahyoid approach	Substernal	Colojejunostomy
10	28/M	Nitric acid	Left coloesophagostomy (antiperistaltic)	Substernal	Colojejunostomy
11	25/M	Hydrochloric acid	Left coloesophagostomy (antiperistatic)	Posterior	Coloesophagostomy + gastro jejunostomy
12	18/F	Sulfuric acid	Left coloesophagostomy (antiperistatic)	Posterior	Coloesophagostomy + gastro jejunostomy
13	22/M	Hydrochloric acid	Left coloesophagostomy (antiperistatic)	Posterior	Coloesophagostomy + gastro jejunostomy
14	25/F	Hydrochloric acid	Left coloesophagostomy (antiperistatic)	Posterior	Coloesophagostomy + gastro jejunostomy
					

In two cases, acute abdominal pain developed the same day after ingestion of the caustic agents and emergency surgery was performed. Because of extensive gastric necrosis in these cases, total gastrectomy and blunt esophagectomy without thoractomy was performed and a left terminal esophagostomy and a feeding jejunostomy completed the procedure. The remaining 12 patients were discharged from the hospital as soon as they could tolerate oral fluids. However, over the following days, dysphagia developed slowly and progressively and the patients returned to hospital. Feeding jejunostomy was performed 8–89 days (mean 48 days) after caustic ingestion. 

Follow-up esophagograms showed severe esophageal stricture requiring the patients to be referred to an endoscopist for antegrade esophageal dilatation. In three cases, due to the long stricture of the esophagus, dilatations were not performed. However, in the remaining cases, dilatations of the esophagus were attempted over 1–3 sessions. As these attempts were unsuccessful, the patients were referred back to our center for definitive surgery. The type of definitive surgery were substernal esophageal bypass in two cases, esophageal resection and replacement of the colon in seven cases and stomach in two cases, and gastroenterostomy in one patient. 

Postoperative follow-up ranged from 5–216 months (mean 53.4 months) among all patients. Two patients died of sepsis postoperatively (on Days 28 and 25, respectively).The cause of sepsis was related to leakage at the proximal anasto- motic site and mediastinitis. In the eight patients who survived, seven postoperative complications developed in five patients (Table. 2). 

**Table 2:** Postoperative complications after esophageal reconstruction for caustic injury

**Case no.**	**Complications**	**Management**	**Outcome**
1	Proximal anastomotic Stricture Regurgitation	Bougienage(twice) Continued Tracheostomy tube for 3 months	Good
2	Salivary fistula	**Conservative**	Good
3	Miss pyloric caustic stenosis* Superaglottic stricture	Extracorporeal gastrojejunostomy&Internal gastrojejunostomy Bougienage &KTP laser & Stening	Good
4	Left subdiaphragmatic Abscess	Surgical drainage	Good
5	Neck Anastomotic fistula	Conservative management	Good

* We missed stenosis of the pylorus due to caustic injury during initial operation

## Discussion

Acid causes coagulation necrosis on tissue contact and it is well known that acids tend to destroy the stomach and spare the esophagus (9,10). This is because of the relative resistance of the squamous epithelium to acid damage and the short duration of contact due to the rapid transit of acid through the esophagus and pyloric spasm (11). Acid injuries more often affect the antrum of the stomach, where antral stenosis results (12). On the other hand, alkaline substances induce liquefaction necrosis, a process that leads to the dissolution of protein and collagen and frequently causes esophageal burns and stricture and only occasionally produce gastric injury (2,10). Corrosive acid ingestion is rare in the West (13), where alkaline substances account for most cases of caustic ingestion (11). In contrast, acid ingestion is common in India where hydrochloric acid is readily available over-the-counter as a cheap toilet cleaner and is the most common corrosive ingested by lower socio-economic groups (1). 

In our series, most caustic accidents involved acids (92.8% of cases) and the most frequently ingested agent was hydrochloric acid (50%). Contrary to the traditional belief that the esophagus is spared in acid ingestion, we found esophageal damage in eight out of 13 patients with acid ingestion (61.5%). Dailwar et al also observed esophageal involvement in 13 out of 15 patients with acid ingestion (13). 

Endoscopy is the best means of assessing the extent and degree of injury as it avoids the occasional missed esophageal burn without oral injuries (14,15). Endoscopy should be performed as soon as the patient’s condition has stabilized and should be performed within 48 hours of the ingestion while the lumen retains its greatest strength (1,4). In our study, endoscopy was performed in one patient on the same day as hospitalization, but the remaining patients underwent endoscopy at a mean time of 16.6 days after injury. 

The management of corrosive injuries of the esophagus and stomach remains controversial. To date there is still no unanimity in the choice of therapy. Traditional treatment by esophagoscopy, steroids and antibiotics as well as the ‘watch, wait and see’ approach for possible full-thickness esophagogastric necrosis remain the mainstay of therapy. However, some practitioners have criticized this policy and advocate an aggressive surgical approach and early immediate laparotomy for patients with at least a second-degree esophageal burn as evidenced by endoscopy and use of intraluminal esophageal stenting for those with second-degree burn or third degree burn without extensive full-thickness necrosis. Immediate esophagogastric resection is recommended for those with extensive full-thickness injury. The most severe complication of esophageal dilatation is esophageal perforation, with an incidence of 1.04% per each dilatation performed. However perforation per se is not an immediate indication for esophageal bypass and patients may be successfully dilated after resolution of the leak (16).

In our series, one patient who underwent antegrade dilatation 3 months after caustic ingestion, experienced a perforation of the esophagus that was treated conservatively, and a surgical procedure was performed later successfully. 

When there is extensive gastric involvement, the esophagus is nearly always necrotic or severely burned, and total gastrectomy and near total esophagectomy is necessary (15). Brune and colleagues advocated esophagectomy by pull-through technique without thoracotomy (17). Emergency blunt esophagectomy is usually easy because of the thrombosis of the periesophageal vessels and lack of adhesion to the mediastinum (18). Two patients in our study required an emergency procedure for acute abdomen and, due to extensive gastric necrosis, total gastrectomy and blunt esophagectomy was performed and a left cervical esophagostomy and a feeding jejunostomy completed the procedure. There was no mortality after emergency procedure. 

A variety of different organs exist for esophageal substitution. The liberal blood supply of the stomach makes it the most reliable organ for use in the intrathoracic replacement of the esophagus (19), but in most cases the stomach is not a suitable candidate for esophageal substitute because it is usually moderately or severely injured by caustic agent (20). When all factors are considered, the order of preference for an esophageal substitute is colon, stomach, then jejunum (15). In our own series we used the colon as a substitute for the esophagus in seven cases, in contrast to the stomach which we were able to use in two cases. Gastroenterostomy was performed in one remaining case with isolated gastric injury after acid ingestion. 

The main controversies in the preparation of a colon interposition are whether to use the right or left colon and the orientation (iso versus antiperistaltic) of the colon interposition. The choice depends in part on the anatomic findings in an individual patient at the time of surgery, but is largely based on the personal preference and training of the surgeon. The majority of investigative and clinical reports suggest that the interposed colon behaves primarily as an inert tube and the passage of materials through it is largely dependent upon gravity (21).

The concomitant development of a severe hypopharyngeal stricture is an infrequent yet critical complication occurring in caustic ingestion. Two cases in our study developed combined hypopharyngo- esophageal stricture, which successfully managed by dilatation and KTP laser and stenting. A study performed by Harlak et al concluded that although with high anastomotic stenosis rates, transhiatal esophagectomy with gastric replacement with cervical anastomosis is a safe procedure which can be performed for the treatment of corrosive esophageal stricture (22).

## Conclusion

Caustic ingestion continues to be a complex clinical challenge and is a dangerous accident. In contrast to children, a suicide attempt is the usual cause of ingestion of corrosive substance in adults.

Although alkaline substances account for most cases of caustic ingestion in Western countries, acid ingestion is more common in our society.

Contrary to the traditional belief that the esophagus is spared in acid ingestion, our study revealed that esophageal damage is common in acid ingestion. Life-threatening perforation and necrosis of the esophagogastric system are best treated by radical surgical resection and delayed reconstruction, while secondary strictures are treated with dilatation and esophageal reconstruction. When combined pharyngeal and esophageal stricture occurs, reconstruction is more difficult and the aim is not only to create a new gullet but also to supply effective respiration and phonation. 

## References

[1] Hugh B, Thomas Kelly D (1999). Michael Corrosive ingestion and the surgeon. J Am Coll Surg.

[2] Wu Ming-Ho, lai Wu-wei (1992). Esophageal reconstruction for esophageal strictures or resection after corrosive injury. Ann Thoracic Surg.

[3] Browne Y Dale, Thompson N James, edited by Charles W, Cummings, John M, Fredrickson, Lee A, Richardson David E Schuller (1998). Caustic ingestion in the book of pediatric otolaryngology head & neck surgery.

[4] Spiegel Joseph R, Sataloff Robert Thayer, Castell DO, Richter JE (1999). Caustic injury of the esophagus in the book of the esophagus.

[5] Thomas Arthur N, Dedo Herbert H, Lim Robert C Jr, Steele Muriel (1976). Pharngoesophageal caustic stricture. Treatment by pharyngogastrostomy compared to colon interposition combined with free bowel graft. Am J Surg.

[6] Doolin Edward J (1994). Composite reconstruction of the Esophagus and hypopharynx after severe caustic injury. Ann Otorhinol Laryngol.

[7] Panieri E, Rode H, Millar AJW, Cywes S (1998). Oesophageal replacement in the management of corrosive strictures: when is surgery indicated?. Pediatr Surg Int.

[8] Postlethwait RW (1983). Colon interposition for esophageal substitution. Surg, Gynecol & Obstet.

[9] Nicosia Jon F, Thornton Joseph P, Folk Frank A, Saletta John D (1974). Surgical management of corrosive gastric injuries. Annals of Surgery.

[10] Estrera Aaron, Taylor Wayne, Millis Lawrence J (1986). Corrosive burns of the Esophagus and stomach: A recommendation for an aggressive surgical approach. Annal Thorac Surg.

[11] Jeng Long Bin Benjamin, Chen Hoang-Yang, Chen Shin - Chen, Hwang Tsann-Long, Jan Yi-Yin, Wang Chia-Siu, Chen Miin-Fu (1994). Upper gastrointestinal ablation for patients with extensive injury after ingestion of strong acid. Arch Surg.

[12] Ashcraft Keith W, Ashcraft Murphy, Sharp Sigalet Synder (2000). Chemicals Esophageal injuries in the book of pediatric surgery.

[13] Dilawari JB, Singh Surjit, Rao PN, Anand BS (1984). Corrosive acid ingestion in man-a clinical and endoscopic study. GUT.

[14] Ferguson Mark K, Marcello Migliore, Staszak M RN, Little Alex G (1989). Early evaluation and therapy for caustic esophageal injury. Am J Surg.

[15] Peters Jeffrey H, Demeester Tom R, edited by Schwartz Shires, Spencer Daly, Fischer Galloway (1999). Caustic injury in the principles of surgery. volume ΙΙ.

[16] Ho-Wu Ming, Lai Wu-Wei, Lin Mu-Yen, Chou Nan-Song (1994). Prevention and management of strictures after hypopharyngocolostomy or esophagocolostomy. Ann Thoracic Surgery.

[17] Brun JG, Koskas F, Dubost C (1984). Blunt thorax oesophageal stripping: Am emergency procedure for caustic ingestion. Br J Surg.

[18] Horvath Ors P, Tiborolah, Gabriella Zentai (1991). Emergency Esophagogastrectomy for treatment of hydrochloric acid. Ann Thorac Surg.

[19] Cebeci Hayrettin, Paksoy Melih, Kaytaz Asim, Unal Ethem (2002). Cololaryngostomy procedure in caustic esophageal burns. Eur J Cardio-Thorac Surg.

[20] Howu Ming, Tseng Yau-Lin, Lin Mu-Yen, Lai Wu-Wei (2001). Esophageal reconstruction for hypopharyn- goesophageal strictures after corrosive injury. European J Cardio-Thoracic Surg.

[21] Ferguson Mark K (2002). the colon as a reconstructive organ In the book of reconstructive surgery of the esophagus edited by Mark K. Ferguson Futura publishing Company inc.

[22] Harlak A, Yigit T, Coskun K, Ozer T, Mentes O, Gülec B, Kozak O (2013). Surgical treatment of caustic esophageal strictures in adults. Int J Surg.

